# Microbiological Analysis in Three Diverse Natural Geothermal Bathing Pools in Iceland

**DOI:** 10.3390/ijerph10031085

**Published:** 2013-03-14

**Authors:** Berglind Osk Th. Thorolfsdottir, Viggo Thor Marteinsson

**Affiliations:** 1 Matis, Department of Food Safety, Environment and Genetics, Vínlandsleið 12, Reykjavík 113, Iceland; 2 School of Engineering and Natural Sciences, University of Iceland, Sæmundargata 2, Reykjavík 101, Iceland

**Keywords:** natural geothermal pool, bacterial count, microbiological parameters, cultured and uncultured bacteria, 16S rRNA gene sequencing

## Abstract

Natural thermal bathing pools contain geothermal water that is very popular to bathe in but the water is not sterilized, irradiated or treated in any way. Increasing tourism in Iceland will lead to increasing numbers of bath guests, which can in turn affect the microbial flora in the pools and therefore user safety. Today, there is no legislation that applies to natural geothermal pools in Iceland, as the water is not used for consumption and the pools are not defined as public swimming pools. In this study, we conducted a microbiological analysis on three popular but different natural pools in Iceland, located at Lýsuhóll, Hveravellir and Landmannalaugar. Total bacterial counts were performed by flow cytometry, and with plate count at 22 °C, 37 °C and 50 °C. The presence of viable coliforms, *Enterococcus* spp. and pseudomonads were investigated by growth experiments on selective media. All samples were screened for noroviruses by real time PCR. The results indicate higher fecal contamination in the geothermal pools where the geothermal water flow was low and bathing guest count was high during the day. The number of cultivated *Pseudomonas* spp. was high (13,000–40,000 cfu/100 mL) in the natural pools, and several strains were isolated and classified as opportunistic pathogens. Norovirus was not detected in the three pools. DNA was extracted from one-liter samples in each pool and analyzed by partial 16S rRNA gene sequencing. Microbial diversity analysis revealed different microbial communities between the pools and they were primarily composed of alpha-, beta- and gammaproteobacteria*.*

## 1. Introduction

Geothermal heat is found in over 700 locations in Iceland [1]. Natural thermal bathing pools are important local resources that have been used by the people in Iceland for centuries. A natural thermal pool is defined as a pool with geothermal water that has not been sterilized, irradiated or treated in any way [2]. There is no legislation that applies to the natural pools in Iceland, so people bathe in them entirely at their own risk. Water for traditional or municipal swimming pools must always fulfill bacteriological water quality criteria and contain a certain chlorine concentration to keep it free from bacteria. However, when the quality of natural thermal pool water is assessed, it does not comply with the criteria for traditional swimming pools. Iceland has not implemented the new EU Bathing Water Directive of 2006.

A vast diversity of microorganisms can be found in pools and other waters used for recreation, originating from different sources, such as humans, animals or the environment. Diseases, some very serious, can be transmitted with bathwater if it is not disinfected before bathing. In many cases such infections have been associated with fecal contamination of the water. Fecal contamination can come from the pool guests’ feces, the water supply itself or from animal feces (such as birds and small animals). Other body fluids (e.g., vomit, mucus, saliva) and flakes of skin are also potential sources of pathogenic bacteria. The best indicators for the assessment of the safety of swimming pool water are disputed. Some researchers emphasize that the microbiological quality of swimming pools is effectively measured by using bacteria that indicate fecal contamination, such as fecal coliforms and enterococci [3]. Others consider that the risk of infection is more associated with microorganisms derived from the skin, mouth, and upper respiratory tract of bathers [3,4]. Therefore, it might be good to detect bacteria from feces as well as other human body fluids.

It should be kept in mind that not all diseases stem from bacteria found in the bathwater itself. Infections can occur due to the natural bacterial flora of the body thriving in response to a reduced immune system following bathing and cooling, so-called “opportunistic pathogens” [5]. Human infection via bathing water can be achieved by direct contact (through cracks in the skin), from air through the respiratory system or directly from the water entering the gastrointestinal tract through the mouth. Studies have shown that while swimming (average 81 min), children absorb an average of 0.63 mL/min of pool water, adult men 0.50 mL/min, and adult women 0.34 mL/min [6].

The public health and safety of natural thermal pools in Iceland has not been monitored on a regular basis. A bacterial analysis was conducted on 1–3 water samples from nine sites in 2002 and the results were presented in a report [2]. The samples were examined for total bacterial count in culture at 37 °C, fecal coliforms, *Pseudomonas aeruginosa* and *Staphylococcus aureus.* Conductivity, turbidity and pH were also measured. Results of the analysis gave some indications of water quality and environmental factors of the sites. The results indicated that there were some differences between locations depending on the guest count and location. A high count of fecal coliforms was measured at one point and time in the Blue Lagoon (800 cfu (colony forming units)/100 mL), Landmannalaugar (550) and several samples from Hveravellir (570–1,300). *Pseudomonas aeruginosa* was also found to be at high count in the Blue Lagoon (200), Laugafell (300), Hveravellir (20,000) and Landmannalaugar (500). The results indicated that “to have a significant picture of the condition of natural pool in Iceland they should be monitored regularly, as their bacterial condition may vary due to man-made changes and changes in the natural environment” [2]. The Public Health Authorities have analyzed water samples from natural pools over the years, but since no regulation exists for these pools, the sampling and measurements have neither been regular nor many.

People are known to bathe in thermal pools in many places around the world, e.g., in France, Germany, Austria, Chechnya, Hungary, Japan, Taiwan, *etc.*, but man-made recreational spas are far more common. Many EU countries have implemented the Bathing Water Directive of 2006, which means they monitor their beaches and inland waters that are used for bathing.

The objective of the study was to assess the microbiological status of Icelandic natural thermal bathing pools by evaluating their microbial diversity by partial 16S rRNA gene sequencing; by comparing total cell count performed by flow cytometry to total viable plate count analysis (at 22 °C, 37 °C and 50 °C); and finally by testing for the presence of viable coliforms, intestinal enterococci, *Pseudomonas aeruginosa* using selective media and noroviruses by real time PCR to estimate the relation between fecal contamination, guest count and water flow of the pools.

**Figure 1:**
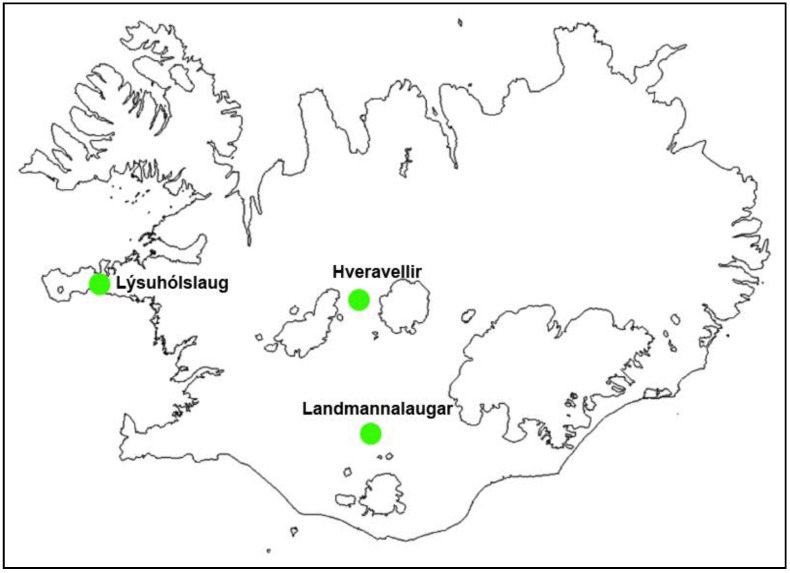
Locations of the study areas in Iceland.

## 2. Materials and Methods

### 2.1. Study Areas

The study areas were three physically different natural pools (Figure 1): Hveravellir in the western part of the highlands (630 m above sea level), Landmannalaugar (500 m.a.s.l.) in the southern part of the highlands, and Lýsuhólslaug in the western part of Iceland (lowland). The natural pool at Hveravellir is made up of stones, located at a high temperature geothermal area where the water is piped a short way from a hot spring. The pool at Landmannalaugar was constructed by damming a big lake that flows from underneath the lava-rock in a low temperature area. Lýsuhólslaug is a completely man-made concrete pool, where the water is piped a fairly long way from a drilled well in the low temperature area. The pools are all operated during the summer and are attended by both foreign and domestic travelers. During the high tourism season, the number of guests is about 40,000 in Hveravellir, 100,000 in Landmannalaugar and 5,000 in Lýsuhólslaug.

### 2.2. Sample Collection, Filtration, Cultivation and Confirmation

Water samples were collected from Lýsuhólslaug on 17 July 2011 (Sunday), 14 August 2011 (Sunday) from Hveravellir, and 24 August 2011 (Wednesday) from Landmannalaugar. Sterilized 5-liter plastic bottles were used and five samples were collected at 20 cm below surface over a 7–13 h period. Temperature, pH and guest count were also recorded. The samples were kept in a cooling box during the day of the sample collection and kept at 4 °C overnight before they were processed (12–24 h after sampling, due to the distant geographical position of the natural pools).

The water samples were quantitatively analyzed for the microbiological parameters of total bacterial count at 22 °C, 37 °C and 50 °C, intestinal enterococci, *E. coli* and *Pseudomonas aeruginosa*. The media used in the procedure were prepared according to the instruction of the manufacturers. Samples of different size (100, 10, 1 and 0.1 mL) were filtered through 0.22 µm membrane filter and the filter placed on the appropriate agar medium; total bacterial count on R2A (Difco R2A agar REF 218263, incubated 2–5 days), *E. coli* on mFC agar (Difco, REF 267720) incubated at 44 °C for 24 h, and confirmed in lactose broth (Oxoid, CM0137) after 24 h incubation at 44 °C, intestinal enterococci on Slanetz & Bartley agar (Oxoid, CM0377) incubated at 37 °C for 48 h and confirmed on Bile aesculin agar (Oxoid, CM0888) after 2 h incubation at 44 °C, *P. aeruginosa* on CNA (Oxoid Pseudomonas agar base CM0559), incubated for 48 h at 37 °C, and confirmed in acetamide broth (Merck, 1.01394) incubated for 24 at 37 °C. 1–2 drops of Nessler’s reagent was then added. One liter of each sample was filtrated for DNA extraction, 16S rDNA amplification and analysis.

### 2.3. Flow Cytometry

Five mL of water from each sample were frozen in 120 µL of 5% glutaraldehyde at −80 °C for cell count. All samples were analyzed on a BD FACS ARIA^TM^ II (BD Biosciences, CA, USA). Samples (300 µL) were mixed with 3 µL of 1/100 diluted SYBR Green and incubated for 20 min at room temperature. Twenty-five µL of counting beads (CountBright^TM^ absolute counting beads from Invitrogen, Life Technologies, Carlsbad, CA, USA) were then put in the mix before they were placed in the flow cytometer. Samples flowed through the cytometer for 30 s (elapsed time) and then events were recorded and counted for 1 min. The BD FACSDiva^TM^ Software (BD Biosciences, San Jose, CA, USA) was used for controlling the instrument and recording data.

### 2.4. Real-Time PCR for Norovirus

Real-time polymerase chain reaction for norovirus genotype 1 was performed for the last sample taken in each pool. Viral RNA was extracted from 140 mL of pre-filtered and treated sample using a QIAamp Viral RNA Mini Kit (Qiagen Inc., Germantown, MD, USA), according to the manufacturer’s instructions. All real time PCR reactions were performed using Brilliant II QRT-PCR 1-Step Master Mix (Agilent Technologies, CA, USA) and MX3005p real time PCR machine (Agilent Tecnologies, Palo Alto, CA, USA). Norovirus GI real time RT-PCR detection was performed using 5 μl of viral RNA template in 25 μl reaction volume, 600 nM of each primer (QNIF4 and NViLCR) and 200 nM probe (TM9). After an initial RT step of 30 min at 50 °C the reaction mixture was incubated at 95 °C for 10 min followed by 40 cycles of 95 °C for 30 s, 50 °C for 30 s and 72 °C for 30 s, finishing with a final extension step at 72 °C for 7 min. Both undiluted and 10× diluted samples were PCR amplified along with a positive GI control (RNA isolated from a contaminated fecal sample).

### 2.5. DNA Extraction

For the extraction of total DNA from the pool water, 1 liter of the last collected sample (taken at the end of the day) was filtered through a sterile gridded 0.45 µm pore size cellulose membrane filter (Millipore Corporation, Bedford, MA, USA) to capture microbial cells. Isolation of chromosomal DNA was carried out by the KingFisher^®^ method (Thermo Scientific, Waltham, MA, USA).

### 2.6. Bacterial 16S rRNA Gene Clone Libraries and Sequencing

Extracted DNA was PCR-amplified in a DNA engine, Terad^®^2 Peltier Thermal Cycler (Bio Rad, Hercules, CA, USA), using the primer pair 341F (5′-CCTACGGGNGGCWGCAG-3′) [7] and 1046R (5′-CGACAGCCATGCANCACCT-3′) [8]. A solution of 2 μL isolated DNA was used with 23 μL of the mix (Buffer GC, enhancer, dNTP (10 mM), 341F, 1046R, enzyme (Taq polymerase) and dH_2_O). The PCR reaction was performed as follows: 5 min of denaturation at 95 °C followed by 35 thermal cycles performed for 30 s at 95 °C, 40 s at 50 °C and 1 min at 68 °C, finishing with a final extension step at 68 °C for 5 min. The PCR product was then incubated at 4 °C until collected.

The amplification products were analyzed by electrophoresis on a 1.0% agarose gel stained with ethidium bromide (3 μL/100 mL). Specific PCR products were cut from the gel under UV-light and isolated from the agarose gel using the Illustra^TM^ GFX^TM^ PCR DNA and Gel Band Purification Kit **(**GE Healthcare**,** Eindhoven, The Netherlands), according to the manufacturer’s instructions. Adenine (A) was first added to the 3′ end of the GFX product. The PCR products from the biomass were then cloned directly by the TA cloning method by using a TOPO TA cloning kit according to the instructions of the manufacturer (Invitrogen, Life Technologies). Plasmid DNA from single colonies was isolated and 1046R reverse primer was used to amplify the 16S rDNA insert before sequencing on an ABI 377 DNA sequencer (Applied Biosystems^®^, Life Technologies, Foster City, CA, USA) by using a Big Dye Terminator Cycle Sequencing Ready Reaction Kit (Applied Biosystems^®^) [9].

### 2.7. Sequencing of 16S rDNA from Pseudomonas Strains

DNA extraction of putative *P. aeruginosa strains* was performed using the Chelex DNA extraction method: 200 μL of 5% Chelex was placed in four eppendorf tubes along with a loop of colonies. The tubes were vortexed, placed in 55 °C for 15 min and vortexed again. The samples were then boiled for 10 min and placed on ice for 3 min. The samples were then centrifuged (11,000 g) for 7 min. The supernatant was removed and later PCR amplified. PCR amplification and 16S rRNA gene sequencing was then performed as described in sections 2.5 and 2.6 without the cloning process.

### 2.8. Phylogenetic Analysis

Sequences were analysed and edited by using the program Sequencer 4.8 from ABI. Both clones and *Pseudomonas* isolates sequences were grouped into operational taxonomic units (OTUs) at a threshold of 98% sequence identity and then aligned by using ClustalW within the MEGA package, version 5.1 [10]. In order to check for the occurrence of cultured species and to confirm *Pseudomonas* spp. isolates, sequences were searched against those deposited in GenBank, through the NCBI BLAST [11,12]. Neighbor-joining phylogenetic trees were constructed with MEGA 5.1 [10] using a representative sequence from each OTU and related GenBank sequences.

## 3. Results

### 3.1. Indicator Bacteria, Total Bacterial Count and Total Cell Count Estimated with Flow Cytometry

The numbers of colonies that grew on selective media to cultivate *E.* *coli, Enterococcus* spp. and *P.* *aeruginosa* are summarized in Table 1. The number of each indicator bacteria refers to bacterial counts in 100 mL of sample. The flow rate of water into the pools and the pool temperature varied between pools. The flow rate into Lýsuholslaug was about 3.8 L/s and the temperature of samples ranged from 32.5 °C to 33.1 °C. The flow rate into Hveravellir was about 2 L/s and the temperature of samples was 33 °C to 42 °C. The flow rate into Landmannalaugar was about 50 L/s and the temperature of samples ranged from 39 °C to 46 °C. The results for total bacterial counts on R2A agar (cultivated at 50 °C, 37 °C and 22 °C) are also summarized in Table 1. Total bacterial count refers to the number of cfu in 1 mL of sample. The last three rows present the results of the total cell counts by flow cytometry and are expressed as the number of microbes in 1 mL of sample. The numbers increased in all pools in relation to time of the day and numbers of bathing guests although some fluctuations during the day were observed.

**Table 1:** Numbers of *Escherichia coli*, *Enterococcus* spp. and *Pseudomonas aeruginosa*, sampling time, temperature, pH, guest count, total bacterial counts at cultivation and total cell counts by flow cytometry in samples from each pool.

	Lýsuhólslaug 17 July 2011					Hveravellir 14 August 2011					Landmannalaugar 24 August 2011				
Sample	1	2	3	4	5	1	2	3	4	5	1	2	3	4	5
Time	13:10	14:45	16:25	18:30	20:00	08:04	12:20	15:30	18:00	20:50	09:44	12:25	14:55	17:45	20:14
Temp (°C)	32.5	32.7	33.1	32.9	32.6	33	38	36	37	42	36	36	36	38	46
pH	7	7	7	7	7	9.5	9.5	9.5	9.5	9.5	6.5	6.6	6.7	6.6	6.7
Guest count^1^	0	9	6	6	0	0	9	2	2	3	0	12	18	24	22
*E. coli ^2,3^*	5	12	7	14	19	220	100	90	150	440	3	5	11	3	4
*Enterococcus ^2,3^*	1	1	3	0	2	47	56	46	81	130	2	1	1	2	3
*P. aeruginosa ^2,3^*	-	-	-	-	-	3,000	12,000	40,000	28,000	30,000	1,400	1,800	2,300	1,300	1,900
Grown at 50 °C ^4^	2	0	400	1	10	290	1,200	2,000	2,500	1,070	830	720	800	770	550
Grown at 37 °C ^4^	1,500	850	800	1,500	1,070	380	1,550	1,400	1,500	1,650	1,200	910	680	780	700
Grown at 22 °C ^4^	2,100	1,500	2,000	2,400	535	135	90	155	150	205	790	780	680	630	740
Bacteria ^5^	2.6 × 10^6^	1.9 × 10^6^	7.5 × 10^5^	5.4 × 10^5^	4.9 × 10^5^	1.6 × 10^5^	1.2 × 10^5^	2.7 × 10^5^	1.7 × 10^5^	3.2 × 10^5^	1.1 × 10^5^	1.2 × 10^5^	1.4 × 10^5^	1.1 × 10^5^	1.3 × 10^5^
Algae/Larger particles ^5^	1.5 × 10^6^	2.0 × 10^6^	9.2 × 10^5^	9.2 × 10^5^	8.7 × 10^5^	8.7 × 10^4^	5.5 × 10^4^	4.0 × 10^5^	2.3 × 10^5^	1.3 × 10^5^	2.9 × 10^4^	5.4 × 10^4^	6.4 × 10^4^	3.3 × 10^4^	3.2 × 10^4^
Total cell count ^5^	5.3 × 10^6^	5.7 × 10^6^	2.8 × 10^6^	2.8 × 10^6^	2.8 × 10^6^	4.2 × 10^5^	3.2 × 10^5^	8.9 × 10^5^	5.8 × 10^5^	6.4 × 10^5^	2.3 × 10^5^	2.6 × 10^5^	3.0 × 10^5^	2.4 × 10^5^	2.5 × 10^5^

^1^ In the pool during sampling. Total guest count on sampling day: Lýsuhólslaug: 79, Hveravellir: approx. 100 and Landmannalaugar: approx. 200.^ 2^ Plate counts (cfu/100 mL). ^3^ *E. coli* and *Enterococcus* confirmed as described earlier, *P. aeruginosa* unconfirmed. ^4^ Total bacterial plate counts on R2A medium (cfu/mL). ^5^ FACS cell count/mL. ^(−)^ Not measured in this pool.

**Figure 2:**
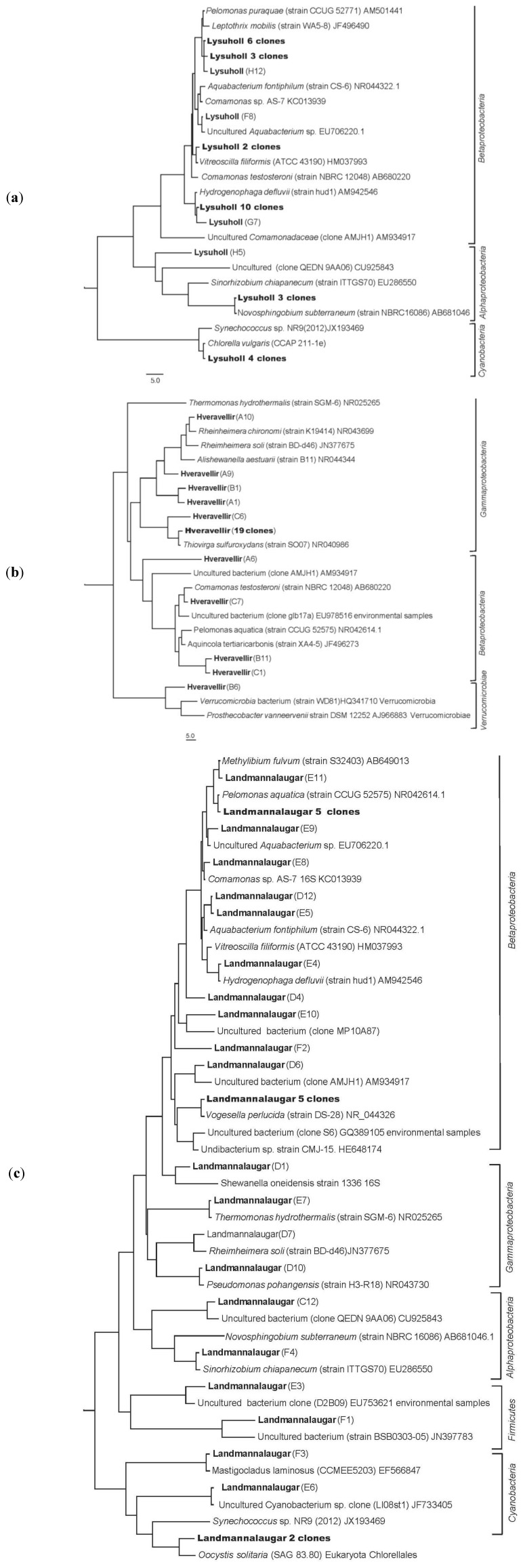
(**a**) Phylogenetic tree based on neighbor-joining analysis of the 16S rRNA gene sequences of 32 bacterial clones from Lýsuhólslaug; (**b**) 29 bacterial clones from Hveravellir; (**c**) 32 bacterial clones from Landmannalaugar. *Aquifex* bacteria were used as an outgroup in all the trees. The scale bar represents the expected % of substitutions per nucleotide position. All OTUs from samples are marked in bold for each clone and their numbers if more than one. The GenBank accession numbers of reference sequences are given for each OTU.

### 3.2. 16S rRNA Gene Sequencing

In total, 16S rRNA genes from 93 clones were sequenced and about 500–630 base pairs (bp) of each clone was used for phylogenic analysis after omitting unreadable bp in sequences. The majority of the clones or OTUs could be affiliated to Proteobacteria (alpha, beta and gamma subgroups) and about 25% (22 OTUs) showed less than 97% similarity with any known type strain, thereof 3 clones that could not be affiliated into specific genera. Figure 2(a), (b), (c) show neighbour-joining trees of sequences from the 16S rRNA clone libraries of each pool. The scale bar represents the expected % of substitutions per nucleotide position and *Aquifex* bacteria were used as an outgroup. The clusters of clones in each tree represent clones with >99% sequence similarity. GenBank accession numbers are given for each reference strain or OTU.

The phylogenetic tree for Lýsuhólslaug (Figure 2 (a)) shows that the most abundant groups are similar to *Pelomonas puraquae* and *Leptothrix mobilis* species (nine clones)*, Hydrogenophaga defluvii* (10 clones), *Novosphingobium subterraneum* (three clones) and some chloroplasts *Chlorella vulgaris* (four clones). One clone could not be affiliated to any genus.

The phylogenetic tree for Hveravellir (Figure 2 (b)) shows that the most abundant group showed high similarity to the species *Thiovirga sulfuroxydans* (19 clones). Most of the other clones could not be affiliated to any known genus, except for *Comamonas* and *Rheinheimera*.

The phylogenetic tree for Landmannalaugar (Figure 2 (c)) shows very high diversity of OTUs in the samples analysed. The OTUs belong to five phyla (beta-, gamma- and alphaproteobacteria, Firmicutes and Cyanobacteria) and most of them could be affiliated with high similarity to known genera. About six OTUs could not be affiliated to known genus, but four of them to uncultured environmental clones.

**Figure 3:**
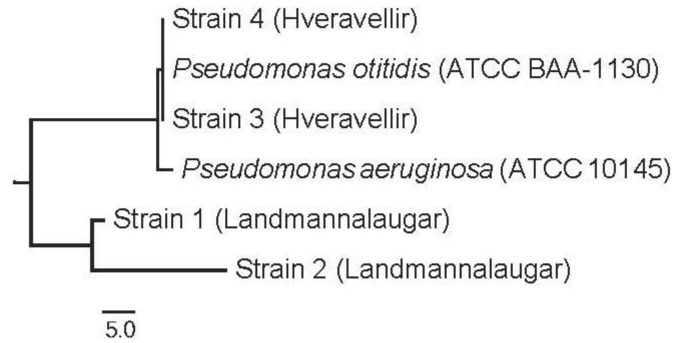
Phylogenetic tree based on neighbor-joining analysis of the 16S rRNA gene sequences of 4 putative *Pseudomonas* strains. The 16S rRNA sequence of *Aquifex* was used as an outgroup. The scale bar represents the expected % of substitutions per nucleotide position.

### *Pseudomonas* Strains

The confirmation of putative *Pseudomonas aeruginosa* strains by Nessler’s reagent was not definitive. Therefore, the 16S rRNA genes from the positive cultured isolates were sequenced for further inspection and to confirm that the strains belonged to the *Pseudomonas* genus and to what extent to *P. aeruginosa*. Isolates 1 and 2 were isolated from Landmannalaugar and isolates 3 and 4 from Hveravellir. The isolates came from samples 4 and 5 in both locations. The phylogenetic tree (Figure 3) shows that isolates 3 and 4 have 100% similarity to *P. otitidis* and 99% similarity to *P. aeruginosa.* Isolates 1 and 2 are more distantly related.

### 3.3. Norovirus Detection in Samples

Norovirus was not detected with the method used in this study in any of the samples collected from the three natural pools.

## 4. Discussion

The study revealed that the three pools meet the microbiological requirements of the Directive no. 2006/7/EC on quality of bathing water in inland areas and had an excellent water quality with respect to *E. coli* (<500 cfu/100 mL) and *Enterococcus* (<200 cfu/100 mL) criteria. However, these limits are quite high and it is of concern whether they should be transferred to the Icelandic natural thermal pools. The physiological conditions of most natural pools are very good for bacterial proliferation and excessive inoculation of pathogens into the pools from humans could cause health problems. According to the guideline given in the Icelandic regulations for the prevention of water pollution no. 796/1999 for recreational waters, both Lýsuhólslaug and Landmannalaugar meet the requirements “I” in the regulation, which means they have little or no fecal contamination. The pool at Hveravellir exceeds requirements “III and IV” in the regulation which indicates some to high fecal contamination. Outside of Europe the limits are lower, e.g., the U.S. Environmental Protection Agency has since 1986 set the limit to 126 cfu/100 mL for *E. coli* and 33 cfu/100 mL for *Enterococcus* [13]. Our study is in agreement to the study performed in 2002, where the public seems to affect the quality of the water, but not enough to become harmful to others pool guests [2]. The unequal numbers of bacteria in the pools is most likely due to different total volume of the pools, water flow, the number of pool guests and the cleaning of the pools. However, it can be asserted that the tourist burden on the pool at Hveravellir had reached its maximum capacity on the day sampling took place. Interestingly but not surprisingly, the numbers of bacteria that grew at 55 °C were higher or at least 9 to 17 times in average higher in the more natural pools at Landmannalaugar and Hveravellir, respectively, than at Lýsuhólslaug. These results go into the opposite direction for the bacteria that grew at 22 °C as the numbers of bacteria in Lýsuhólslaug were 2 to 11 higher than in average higher than at Landmannalaugar and Hveravellir, respectively. This difference may be explained by the lower temperature measured at Lýsuholslaug, stimulating the growth of bacteria tolerating better lower temperatures compared to the other pools and vice versa. The number of bacteria that grew at 37 °C was similar in all pools. The flow cytometry technique identified more cells than were measured by the cultivation methods employed in the present study. This suggests that nonculturable bacteria were present in the sample, or in a nonculturable state. That correlates with the theory that only <1–5% of microorganisms can be grown by conventional methods [9]. It should be noted that the total bacterial counts in this study should not be taken as absolute values as only one count represents each sampling point. Therefore we observe here an increasing trend for the number of cells with time and bath guest but its statistical significance is not demonstrated. The guests count on sampling days were accurate for Lýsuhólslaug (79), but estimated in Hveravellir (100) and Landmannalaugar (200).

In this study, DNA was isolated from all microorganisms (whether they were alive or not) in the samples. This will include bacteria that were not cultured on the media used, either due to their unculturable state or unsatisfied growth requirements. Sequencing of 16S rRNA genes from the last sample collected from each pool (32 clones) was performed. A total of 93 clones were successfully sequenced and about 500–700 bases of each gene were retrieved. These sequences or OTUs showed homology to alpha-, beta- and gammaproteobacteria, Cyanobacteria, and interestingly also from eukaryotic cells. This suggests that free floating chloroplasts that contain 16S rRNA genes were released from eukaryotic algal cells but these cells were also detected in high numbers with flow cytometry. Most of the OTUs could be affiliated to bacterial species that have been isolated or detected before, in hot springs or pools, and some in water and soil. No pathogens were detected by this method. Interestingly, many of the OTUs detected in the samples were not closely related to bacteria that have been cultivated. The OTUs’ profiles obtained differed among the three pools but three genera were found in more than one pool: *Pelomonas, Hydrogenophaga* and *Rheinheimera*. Overall, the range of biodiversity in the pools was very different, which could be explained by the environmental conditions; pH, conductivity and concentrations of chemicals (e.g., silica) are different for each pool [14,15,16].

All samples contained bacterial strains that were grown on the culture medium specific for *P. aeruginosa.* Many colonies were fluorescent, but confirmation with formation of ammonia from cultures of acetamide broth was not conclusive. The change of color should be from a light yellowish color to bright yellow or red-brown color, but the changes showed a paler yellow color than the unambiguous confirmation of *P. aeruginosa* should show*.* Therefore, four putative *Pseudomonas* isolates were selected for sequencing of 16S rRNA genes to confirm our color test. The results showed that strains from Hveravellir had 100% gene similarity to *P. otitidis*, which can cause ear infection (otitis) and was first described in 2006 [17]. These strains also had a 99% gene similarity to *P. aeruginosa,* and BLAST searches revealed that strains also had a 99% gene similarity to *P. alcaligenes, P. mosselii and P. guezennei.* The *Pseudomonas* strains from Landmannalaugar were more distantly related to these species. All these *Pseudomonas* species, except for *P. guezennei*, are opportunistic pathogens, but it is not known whether the strains in this study have virulence factors. Nevertheless, according to Icelandic regulation (No. 814/2010), operators of swimming pools must screen for *P. aeruginosa*, and there is zero tolerance for such bacteria in bathwater.

The OTUs that were 99–100% homogenous to *Hydrogenophaga* and *Thiovirga* spp. were the most abundant in the samples from Lýsuhólslaug and Hveravellir. The diversity was especially low in Hveravellir as one taxon was dominating the sample with *Thiovirga* spp. It would be necessary to sequence more clones or to perform deep sequencing to detect underrepresented or rare microorganisms [7]. No pathogens were detected by sequencing in this study, but the screening of *E. coli, Enterococcus* spp. and *Pseudomonas* spp. by selective culture media revealed that their number can become quite large when the guest count in the natural pools is high and water flow is too low to renew the water sufficiently.

The water in Landmannalaugar flows at a high rate (50–75 L/s) and the renewal rate of the water is fast compared to the size of the pool (about 42 min). It can therefore be assumed that bacterial contamination is low in spite of numerous visitors. Interestingly, the pool contained similar numbers of bacteria that grew at 50 °C, 37 °C and 22 °C, despite its rather high water temperature. At Hveravellir the flow is low (about 1–3.5 L/s, that comes in spurts) and a large number of visitors generates a lot of stress on the pool environment. The pool capacity is about 2.1 tonnes of water and the renewal time is about 6 h. The number of *E. coli, Enterococcus* spp. and *Pseudomonas* spp. was highest in this pool. Moreover the pool contained the highest number of bacteria that grew at 50 °C and 37 °C but the lowest at 22 °C, which may be related to the high water temperature. The flow is also quite low (3.8 L/s) in Lýsuhólslaug, and it takes about 5 h to renew the water in that pool. However, all visitors must shower first before entering the pool, which decreases the spike from human bacterial flora into the pool. It therefore has a good effect on the microbiological quality of the water as we have observed in this study. Nevertheless the pool contained the highest number of bacteria that grew at 37 °C and 22 °C, but lowest at 50 °C as could be expected from the lower water temperature.

Our findings suggest that the quality of bathing water depends on the interaction of guest count, pool size, flow rate and the hygiene of pool guests. The hygiene part is important because guests carry dirt on their skin and swimwear to the water (e.g., residues from urine and feces, skin particles, *etc.*). It is therefore important that bathers wash thoroughly before entering the pools. It is also very important that people stay away from the pools after illnesses (e.g., diarrhea). Very young children, elderly and people with impaired immune systems should avoid natural pools because of the fecal contamination that may be present in the pool and *P. otitidis* that can cause ear infection. It cannot be ruled out that *P. aeruginosa* is present in natural pools, which can cause a variety of other infections such as pneumonia, septic shock, ear, urinary tract, gastrointestinal and skin infections [18].

Health authorities report very few infections associated with natural pools. This could be a fact, so there are no problems associated to natural pools, or the problem could be hidden. The bathers might not link their illness to their visit to a natural pool or seek medical help due to symptoms. If tourists in Iceland get an infection (e.g., skin, respiratory, gastrointestinal or urinary tract) during their travel around the country, it can become difficult to trace its source. This is particularly the case for foreign tourists staying in the country for a few days, doing various outdoor activities, and tasting various kinds of food. The infection might not even be detected until they get home.

The indicator bacteria *E. coli* and *Enterococcus* indicate that fecal contamination is present, and thus potential contamination by other intestinal bacteria. The pools at Hveravellir and Landmannalaugar are probably the most visited natural thermal pools in Iceland (including the Blue Lagoon and the beach at Nauthólsvík), so there is reason to believe that microbial contamination will not become greater in other pools in the country.

Our results indicate that it is unlikely that humans are at risk for norovirus infection in the natural pools, although individual cases might occur, as in many conventional swimming pools abroad. However, our sampling was only performed once in each pool, which is not enough for any speculation on the possible presence of noroviruses, and more thorough studies will be needed.
